# From a Sister State

**Published:** 1894-02

**Authors:** 


### FROM A SISTER STATE.

Under the title, "TWO DOCTORS," our esteemed neighbor, the *Alabama Medical and Surgical Age*, thus comments on the late defeat of the examining bill:

Be it said to the shame and discredit of the two physicians who were members of the last session of the Georgia legislature, that they were mainly responsible for the defeat of the bill which was before that body of lawmakers to regulate the practice of medicine in the State of Georgia. THE ATLANTA MEDICAL AND SURGICAL JOURNAL informs us that the bill was referred to these two doctors, and they managed to kill it, or prevent its becoming a law. Acting as politicians, we might expect some such result; or if seeking some political end, we could more readily understand the position taken by these gentlemen; but when entrusted with so important a matter as the passage of this bill would be to the whole people of Georgia, we confess our astonishment at the disposition made of it. The bill was defeated. This was an opportunity two physicians had to protect the poor and ignorant of Georgia's population from incompetent practitioners of medicine, and honor themselves and the medical profession of Georgia, but they chose to do neither. So Georgia, noted as the Empire State of the South, will still be a fertile field for medical quacks and incompetent medical men, and the poor and ignorant of her population will suffer the consequences. There is no State in the Union which needs a medical examining board more than the State of Georgia, and we hope to see the day when the leading medical men of that State, who are working so persistently, will succeed in their laudable efforts, and all who practice medicine in the State will have to stand an examination before they will be allowed to hang out their shingle.

---

FROM the daily papers we learn that Dr. William Moor, of New York, has discovered that permanganate of potassium is an antidote to morphine poisoning. To test his discovery Dr. Moor is said to have taken three grains of morphine in the presence of several physicians. He then drank a solution of four grains of permanganate of potassium in four ounces of water. No sign of poisoning was noticeable. If the account be true the discovery is one of great importance.

